# Supplementary material for: Discovery and application of insertion-deletion (INDEL) polymorphisms for QTL mapping of early life-history traits in Atlantic salmon
Source: BMC Genomics. 2010 Mar 8;11:156. doi: 10.1186/1471-2164-11-156 (PMC2838853; doi:10.1186/1471-2164-11-156)
Supplement: Additional file 2 — Information on developed 76 locus single-run INDEL panel in Atlantic salmon. Information on fluorescence labeling, primer concentrations, PCR pooling and links to alignments, INDEL motifs and GENESCAN (Burge and Karlin 1997) predictions of genes/exons are available in html format. [file 1471-2164-11-156-S2.ZIP › Additionalfile2/table10789.html]

Contig Alignment

```
Base	A B C D E   
1	. G . . .    
2	. G . . .    
3	. G . . .    
4	. G . . .    
5	. C . . .    
6	. T . . .    
7	. G . . .    
8	. A . . .    
9	. A . . .    
10	. A . . .    
11	. A . . .    
12	. C . . .    
13	. A . . .    
14	. T . . .    
15	. C . . .    
16	. C . . .    
17	. A . . .    
18	. C . . .    
19	. A . . .    
20	. A . . .    
21	. G . . .    
22	. G . . .    
23	. A . . .    
24	. C . . .    
25	. T . . .    
26	. A . . .    
27	. T . . .    
28	. G . . .    
29	. T . . .    
30	. C . . .    
31	. T . . .    
32	. T . . .    
33	. C C . .    
34	. A A . .    
35	. A A . .    
36	. A A . .    
37	. C C . .    
38	. T T . .    
39	. G G . .    
40	. C C . .    
41	. A A . .    
42	. T T . .    
43	. T T . .    
44	. T T . .    
45	. C C . .    
46	. A A . .    
47	. A A . .    
48	. A A . .    
49	. T T . .    
50	. C C . .    
51	. C C . .    
52	. C C . .    
53	. A A . .    
54	. C C . .    
55	. G G . .    
56	. T T . .    
57	. C C . .    
58	T T T . .    
59	A A A . .    
60	C C C . .    
61	T T T . .    
62	A A A . .    
63	C C C . .    
64	T T T . .    
65	T T T . .    
66	C C C . .    
67	A A A . .    
68	G G G . .    
69	A A A . .    
70	T T T . .    
71	C C C . .    
72	T T T . .    
73	G G G . .    
74	A A A . .    
75	G G G . .    
76	A A A . .    
77	G G G . .    
78	T T T . .    
79	G G G . .    
80	A A A . .    
81	A A A . .    
82	T T T . .    
83	A A A . .    
84	C C C . .    
85	A A A . .    
86	C C C . .    
87	A A A . .    
88	T T T . .    
89	T T T . .    
90	T T T . .    
91	G G G . .    
92	A A A . .    
93	G G G . .    
94	A A A . .    
95	G G G . .    
96	G G G . .    
97	T T T . .    
98	G G G . .    
99	G G G . .    
100	A A A . .    
101	T T T . .    
102	G G G . .    
103	G G G . .    
104	A A A . .    
105	G G G . .    
106	G G G . .    
107	T T T . .    
108	C C C . .    
109	A A A . .    
110	T T T . .    
111	T T T . .    
112	C C C . .    
113	G G G . .    
114	C C C . .    
115	A A A . .    
116	G G G . .    
117	T T T . .    
118	G G G . .    
119	C C C . .    
120	C C C . .    
121	A A A . .    
122	C C C . .    
123	A A A . .    
124	G G G . .    
125	T T T . .    
126	C C C . .    
127	T T T . .    
128	C C C . .    
129	T T T . .    
130	G G G G .    
131	C C C C .    
132	C C C C .    
133	A A A A .    
134	G G G G .    
135	C C C C .    
136	A A A A .    
137	G G G G .    
138	C C C C .    
139	A A A A .    
140	C C C C .    
141	C C C C .    
142	C C C C .    
143	G G G G .    
144	C C C C .    
145	A A A A .    
146	T T T T .    
147	T T T T .    
148	C C C C .    
149	T T T T .    
150	G G G G .    
151	A A A A .    
152	G G G G .    
153	C C C C .    
154	C C C C .    
155	G G G G .    
156	G G G G .    
157	A A A A .    
158	A A A A .    
159	A A A A .    
160	G G G G .    
161	A A A A .    
162	G G G G .    
163	C C C C .    
164	C C C C .    
165	C C C C .    
166	C C C C .    
167	A A A A .    
168	T T T T .    
169	C C C C .    
170	C C C C .    
171	G G G G .    
172	T T T T .    
173	A A A A .    
174	C C C C .    
175	T T T T .    
176	G G G G .    
177	A A A A .    
178	T T T T .    
179	C C C C .    
180	C C C C .    
181	A A A A .    
182	A A A A .    
183	A A A A .    
184	G G G G .    
185	A A A A .    
186	G G G G .    
187	C C C C .    
188	A A A A .    
189	A A A A .    
190	G G G G .    
191	G G G G .    
192	G G G G .    
193	C C C C .    
194	T T T T .    
195	C C C C .    
196	T T T T .    
197	C T C C .    
198	T T T T .    
199	C C C C .    
200	C C C C .    
201	C C C C .    
202	T T T T .    
203	G G G G .    
204	T T T T .    
205	C C C C .    
206	T T T T .    
207	C C C C .    
208	C C C C .    
209	A A A A .    
210	T T T T .    
211	G G G G .    
212	C C C C .    
213	C C C C .    
214	T T T T .    
215	C C C C .    
216	T T T T .    
217	T T T T .    
218	C C C C .    
219	T T T T .    
220	C C C C .    
221	C C C C .    
222	C C C C .    
223	T T T T .    
224	A A A A .    
225	C C C C .    
226	C C C C .    
227	A A A A .    
228	C C C C .    
229	A A A A .    
230	A A A A .    
231	T T T T .    
232	C C C C C    
233	T T T T T    
234	C C C C C    
235	C C C C C    
236	C C C C C    
237	A A A A A    
238	T T T T T    
239	A A A A A    
240	G G G G G    
241	G G G G G    
242	G G G G G    
243	T T T T T    
244	C C C C C    
245	T T T T T    
246	G G G G G    
247	G G G G G    
248	C C C C C    
249	C C C C C    
250	A A A A A    
251	C C C C C    
252	A A A A A    
253	T T T T T    
254	C C C C C    
255	T T T T T    
256	A A A A A    
257	G G G G G    
258	C C C C C    
259	C C C C C    
260	T T T T T    
261	C C C C C    
262	T T T T T    
263	C C C C C    
264	A A A A A    
265	T T T T T    
266	C C C C C    
267	A A A A A    
268	C C C C C    
269	A A A A A    
270	C C C C C    
271	T T T T T    
272	A A A A A    
273	C C C C C    
274	G G G G G    
275	A A A A A    
276	C C C C C    
277	T T T T T    
278	C C C C C    
279	C C C C C    
280	C C C C C    
281	C C C C C    
282	A A A A A    
283	A A A A A    
284	G G G G G    
285	C C C C C    
286	A A A A A    
287	G G G G G    
288	C C C C C    
289	A A A A A    
290	A A A A A    
291	G G G G G    
292	C C C C C    
293	T T T T T    
294	G G G G G    
295	A A A A A    
296	A A A A A    
297	G G G G G    
298	T T T T T    
299	A A A A A    
300	G G G G G    
301	A A A A A    
302	G G G G G    
303	C C C C C    
304	C C C C C    
305	C C C C C    
306	C C C C C    
307	T T T T T    
308	C C C C C    
309	T T T T T    
310	G G G G G    
311	G G G G G    
312	C C C C C    
313	G G G G G    
314	C C C C C    
315	C C C C C    
316	A A A A A    
317	C C C C C    
318	A A A A A    
319	T T T T T    
320	T T T T T    
321	C C C C C    
322	A A A A A    
323	C C C C C    
324	T T T T T    
325	T T T T T    
326	C C C C C    
327	A A A A A    
328	C C C C C    
329	T T T T T    
330	T T T T T    
331	C C C C C    
332	T T T T T    
333	C C C C C    
334	T T T T T    
335	G G G G G    
336	G G G G G    
337	A A A A A    
338	C C C C C    
339	A A A A A    
340	C C C C C    
341	T T T T T    
342	C C C C C    
343	T T T T T    
344	T T T T T    
345	G G G G G    
346	A A A A A    
347	T T T T T    
348	T T T T T    
349	T T T T T    
350	C C C C C    
351	C C C C C    
352	T T T T T    
353	T T T T T    
354	T T T T T    
355	T T T T T    
356	C C C C C    
357	T T T T T    
358	A A A A A    
359	C C C C C    
360	A A A A A    
361	C C C C C    
362	A A A A A    
363	T T T T T    
364	A A A A A    
365	T T T T T    
366	C C C C C    
367	T T T T T    
368	G G G G G    
369	G G G G G    
370	G G G G G    
371	A A A A A    
372	C C C C C    
373	A A A A A    
374	T T T T T    
375	A A A A A    
376	T T T T T    
377	G G G G G    
378	T T T T T    
379	A A A A A    
380	C C C C C    
381	A A A A A    
382	T T T T T    
383	T T T T T    
384	G G G G G    
385	A A A A A    
386	G G G G G    
387	C C C C C    
388	A A A A A    
389	G G G G G    
390	T T T T T    
391	G G G G G    
392	T T T T T    
393	C C C C C    
394	C C C C C    
395	A A A A A    
396	T T T T T    
397	G G G G G    
398	C C C C C    
399	A A A A A    
400	C C C C C    
401	A A A A A    
402	A A A A A    
403	T T T T T    
404	C C C C C    
405	T T T T T    
406	T T T T T    
407	G G G G G    
408	G G G G G    
409	T T T T T    
410	G G G G G    
411	C C C C C    
412	T T T T T    
413	T T T T T    
414	T T T T T    
415	T T T T T    
416	T T T T T    
417	A A A A A    
418	A A A A A    
419	T T T T T    
420	G G G G G    
421	C C C C C    
422	T T T T T    
423	T T T T T    
424	T T T T T    
425	A A A A A    
426	A A A A A    
427	T T T T T    
428	G G G G G    
429	G G G G G    
430	T T T T T    
431	G G G G G    
432	A A A A A    
433	C C C C C    
434	A A A A A    
435	T T T T T    
436	G G G G G    
437	T T T T T    
438	C C C C C    
439	C C C C C    
440	G G G G G    
441	C C C C C    
442	T T T T T    
443	G G G G G    
444	G G G G G    
445	A T T T T    
446	. T T T T    
447	. T T T T    
448	. A A A A    
449	. G G G G    
450	. A A A A    
451	. C C C C    
452	. T T T T    
453	. G G G G    
454	. C C C C    
455	. A A A A    
456	. T T T T    
457	. C C C C    
458	. A A A A    
459	. G G G G    
460	. A A A A    
461	. G G G G    
462	. A A A A    
463	. C C C C    
464	. T T T T    
465	. G G G G    
466	. C C C C    
467	. A A A A    
468	. T T T T    
469	. T T T T    
470	. T T T T    
471	. T T T T    
472	. C C C C    
473	. A A A A    
474	. G G G G    
475	. C C C C    
476	. A A A A    
477	. T T T T    
478	. T T T T    
479	. T T T T    
480	. T T T T    
481	. T T T T    
482	. A A A A    
483	. C C C C    
484	. T T T T    
485	. C C C C    
486	. A A A A    
487	. G G G G    
488	. C C C C    
489	. T T T T    
490	. C C C C    
491	. T T T T    
492	. G G G G    
493	. A A A A    
494	. A A A A    
495	. A A A A    
496	. C C C C    
497	. T T T T    
498	. C - - C  2	 indel  
499	. T - - T  2	 indel  
500	. T T T T    
501	. T T T T    
502	. T T T T    
503	. T T T T    
504	. G G G G    
505	. T T T T    
506	. A A A A    
507	. G G G G    
508	. G G G G    
509	. A A A A    
510	. T T T T    
511	. G G G G    
512	. C C C C    
513	. C C C C    
514	. A A A A    
515	. T T T T    
516	. T T T T    
517	. T T T T    
518	. T T T T    
519	. A A A A    
520	. T T T T    
521	. A A A A    
522	. C C C C    
523	. T T T T    
524	. G G G G    
525	. T T T T    
526	. T T T T    
527	. T T T T    
528	. T T T T    
529	. C C C C    
530	. T T T T    
531	. A A A A    
532	. A A A A    
533	. C C C C    
534	. G G G G    
535	. G G G G    
536	. G G G G    
537	. C C C C    
538	. T T T T    
539	. G G G G    
540	. A A A A    
541	. A A A A    
542	. T T T T    
543	. C C C C    
544	. A A A A    
545	. C C C C    
546	. T T T T    
547	. T T T T    
548	. A A A A    
549	. C C C C    
550	. T T T A    
551	. G G G G    
552	. T T T T    
553	. A A A A    
554	. C C C C    
555	. T T T T    
556	. T T T T    
557	. C C C C    
558	. T T T T    
559	. G G G G    
560	. G G G G    
561	. T T T T    
562	. T T T T    
563	. T T T T    
564	. C C C C    
565	. A A A A    
566	. C C C C    
567	. A A A A    
568	. T T T T    
569	. T T T T    
570	. C C C C    
571	. T T T T    
572	. G G G G    
573	. T T T T    
574	. C C C C    
575	. A A A A    
576	. T T T T    
577	. T T T T    
578	. G G G G    
579	. G G G G    
580	. A A A A    
581	. T T T T    
582	. T T T T    
583	. G G G G    
584	. T T T T    
585	. T T T T    
586	. T T T T    
587	. T T T T    
588	. T T T T    
589	. A A A A    
590	. T T T T    
591	. G G G G    
592	. G G G G    
593	. T T T T    
594	. G G G G    
595	. G G G G    
596	. G G G G    
597	. A A A A    
598	. A A A A    
599	. C C C C    
600	. A A A A    
601	. T T T T    
602	. G G G G    
603	. T T T T    
604	. T T T T    
605	. G G G G    
606	. A A A A    
607	. T T T T    
608	. A A A A    
609	. C C C C    
610	. A A A A    
611	. G G G G    
612	. A A A A    
613	. G G G G    
614	. A A A A    
615	. T T T T    
616	. T T T T    
617	. C C C C    
618	. T T T T    
619	. G G G G    
620	. C C C C    
621	. A A A A    
622	. T T T T    
623	. T T T T    
624	. T T T T    
625	. T T T T    
626	. T T T T    
627	. A A A A    
628	. A A A A    
629	. A A A A    
630	. C C C C    
631	. T T T T    
632	. T T T T    
633	. G G G G    
634	. T T T T    
635	. T T T T    
636	. T T T T    
637	. T T T T    
638	. T T T T    
639	. T T T T    
640	. T T T T    
641	. C C C C    
642	. A A A A    
643	. T T T T    
644	. A A A A    
645	. G G G G    
646	. A A A A    
647	. T T T T    
648	. G G G G    
649	. G G G G    
650	. T T T T    
651	. T T T T    
652	. A A A A    
653	. G G G G    
654	. A A A A    
655	. A A A A    
656	. T T T T    
657	. A A A A    
658	. T T T T    
659	. C C C C    
660	. A A A A    
661	. C C C C    
662	. T T T T    
663	. G G G G    
664	. C C C C    
665	. A A A A    
666	. T T T T    
667	. C C C C    
668	. C C C C    
669	. T T T T    
670	. C C C C    
671	. A A A A    
672	. A A A A    
673	. G G G G    
674	. A A A A    
675	. T T T T    
676	. T T T T    
677	. T T T T    
678	. G G G G    
679	. T T T T    
680	. G G G G    
681	. T T T T    
682	. C C C C    
683	. T T T T    
684	. C C C C    
685	. C C C C    
686	. T T T T    
687	. T T T T    
688	. G G G G    
689	. T T T T    
690	. T T T T    
691	. C C C C    
692	. A A A A    
693	. T T T T    
694	. T T T T    
695	. T T T T    
696	. T T T T    
697	. A A A A    
698	. T T T T    
699	. G G G G    
700	. G G G G    
701	. G G G G    
702	. G G G G    
703	. G G G G    
704	. A A A A    
705	. A A A A    
706	. A A A A    
707	. A . A A    
708	. T . T T    
709	. A . A A    
710	. A . A A    
711	. T . T T    
712	. A . A A    
713	. A . A A    
714	. T . T T    
715	. T . T T    
716	. A . A A    
717	. A . A A    
718	. A . A A    
719	. A . A A    
720	. . . A A    
721	. . . A A    
722	. . . A A    
723	. . . T T    
724	. . . A T    
725	. . . A A    
726	. . . T T    
727	. . . T T    
728	. . . G G    
729	. . . C C    
730	. . . C C    
731	. . . A A    
732	. . . A A    
733	. . . A A    
734	. . . C C    
735	. . . A A    
736	. . . G G    
737	. . . T T    
738	. . . T T    
739	. . . T T    
740	. . . T T    
741	. . . A A    
742	. . . C C    
743	. . . C C    
744	. . . A A    
745	. . . T T    
746	. . . T T    
747	. . . T T    
748	. . . T T    
749	. . . C C    
750	. . . C C    
751	. . . T T    
752	. . . A A    
753	. . . C C    
754	. . . A A    
755	. . . C C    
756	. . . T T    
757	. . . T T    
758	. . . T T    
759	. . . T T    
760	. . . A A    
761	. . . A A    
762	. . . T T    
763	. . . G G    
764	. . . C C    
765	. . . A A    
766	. . . A A    
767	. . . T T    
768	. . . T T    
769	. . . T T    
770	. . . T T    
771	. . . A A    
772	. . . C C    
773	. . . T T    
774	. . . A A    
775	. . . C C    
776	. . . A A    
777	. . . T T    
778	. . . T T    
779	. . . G G    
780	. . . G G    
781	. . . T T    
782	. . . G G    
783	. . . G G    
784	. . . T T    
785	. . . G G    
786	. . . A A    
787	. . . G G    
788	. . . G G    
789	. . . A A    
790	. . . G G    
791	. . . A A    
792	. . . C C    
793	. . . T T    
794	. . . T T    
795	. . . G G    
796	. . . A A    
797	. . . A A    
798	. . . A A    
799	. . . T T    
800	. . . G G    
801	. . . T T    
802	. . . G G    
803	. . . T T    
804	. . . T T    
805	. . . G G    
806	. . . T T    
807	. . . T T
```
